# Menthal health of internally and externally displaced persons in war period

**DOI:** 10.1192/j.eurpsy.2023.175

**Published:** 2023-07-19

**Authors:** N. O. Maruta

**Affiliations:** Department of borderline disorders, Institution of neurology, psychiatry and narcology of NAMS of Ukraine, Kharkiv, Ukraine

## Abstract

**Abstract:**

Workshop War in Ukraine – a Big Challenge for the Mental Health Care

**
Abstract of presentation Mental health of internally and externally displaced persons in war period (Ukrainian experience)**

Maruta

*“Institute of Neurology, Psychiatry and Narcology of the National Academy of Medical Sciences of Ukraine” State Institution*

The presentation is devoted to mental health problems of internally and externally displaced persons during the war. Issues of etiology, pathogenesis and clinic-psychopathological manifestations of mental disorders in displaced persons are considered.

The main focus is on risk, anti-risk factors and stress coping strategies that prevent the development of mental disorders.

The presentation also provided a system of therapy and rehabilitation for internally and externally displaced persons, as well as an evaluation of their effectiveness.

**Disclosure of Interest:**

None Declared

